# Employment of diverse in vitro systems for analyzing multiple aspects of disease, hereditary hemorrhagic telangiectasia (HHT)

**DOI:** 10.1186/s13578-024-01247-z

**Published:** 2024-05-22

**Authors:** Hyebin Koh, Woojoo Kang, Ying-Ying Mao, Jisoo Park, Sangjune Kim, Seok-Ho Hong, Jong-Hee Lee

**Affiliations:** 1 Futuristic Animal Resource & Research Center (FARRC), Korea Research Institute of Bioscience and Biotechnology (KRIBB), Cheongju, Republic of Korea; 2 Department of Functional Genomics, KRIBB School of Bioscience, Korea University of Science and Technology (UST), Daejeon, Republic of Korea; 3 National Primate Research Center (NPRC), Korea Research Institute of Bioscience and Biotechnology (KRIBB), Cheongju, Republic of Korea; 4 Department of Internal Medicine, School of Medicine, Kangwon National University, Chuncheon, Republic of Korea; 5 Department of Animal Science and Biotechnology, College of Agriculture and Life Science, Chungnam National University, Daejeon, Republic of Korea; 6 Department of Biological Sciences and Biotechnology, Chungbuk National University, Cheongju, Republic of Korea; 7 KW-Bio Co., Ltd, Chuncheon, South Korea

**Keywords:** Hereditary hemorrhagic telangiectasia, *ENDOGLIN*, hPSC modeling, Endothelial cell, Smooth muscle cell, Blood vessel organoid, Inflammatory response, High susceptibility

## Abstract

**Background:**

In vitro disease modeling enables translational research by providing insight into disease pathophysiology and molecular mechanisms, leading to the development of novel therapeutics. Nevertheless, in vitro systems have limitations for recapitulating the complexity of tissues, and a single model system is insufficient to gain a comprehensive understanding of a disease.

**Results:**

Here we explored the potential of using several models in combination to provide mechanistic insight into hereditary hemorrhagic telangiectasia (HHT), a genetic vascular disorder. Genome editing was performed to establish hPSCs (H9) with *ENG* haploinsufficiency and several in vitro models were used to recapitulate the functional aspects of the cells that constitute blood vessels. In a 2D culture system, endothelial cells showed early senescence, reduced viability, and heightened susceptibility to apoptotic insults, and smooth muscle cells (SMCs) exhibited similar behavior to their wild-type counterparts. Features of HHT were evident in 3D blood-vessel organoid systems, including thickening of capillary structures, decreased interaction between ECs and surrounding SMCs, and reduced cell viability. Features of *ENG* haploinsufficiency were observed in arterial and venous EC subtypes, with arterial ECs showing significant impairments. Molecular biological approaches confirmed the significant downregulation of Notch signaling in HHT-ECs.

**Conclusions:**

Overall, we demonstrated refined research strategies to enhance our comprehension of HHT, providing valuable insights for pathogenic analysis and the exploration of innovative therapeutic interventions. Additionally, these results underscore the importance of employing diverse in vitro systems to assess multiple aspects of disease, which is challenging using a single in vitro system.

**Supplementary Information:**

The online version contains supplementary material available at 10.1186/s13578-024-01247-z.

## Introduction

The restricted access to diseased organs in vivo hampers the investigation of aspects of human pathology. To address this, in vitro approaches and models have been developed to recapitulate in vivo pathophysiological and clinical manifestations. Research on pluripotent stem cells (PSCs) has facilitated breakthroughs in disease modeling and drug discovery, thereby advancing cell biology and pathophysiology [1]. Patient-specific induced PSCs (iPSCs) carrying disease-causing genetic mutations and genome-editing technology (such as CRISPR/Cas9, TALENs, and ZFNs) enable the precise introduction of disease-related genetic mutations, thus facilitating the generation of in vitro disease models [2]. Endeavors in differentiation techniques allow the generation of cell types of interest, including pancreatic beta cells, cardiomyocytes, and neural cells, enabling in vitro modeling of diabetes, hypertrophic cardiomyopathy, and neurodegenerative conditions such as Parkinson’s disease [3, 4]. The generation of pure populations of endothelial cell (EC) subtypes, specifically arterial and venous ECs, allows the mapping of preferential targets of Nipah and Hendra viruses, which exhibit arterial tropism [5]. Although 2D models have enabled functional analyses of various cell types, they have drawbacks for evaluating in vivo cell–cell and cell–extracellular matrix (ECM) interactions, as well as the functions of these interactions in diseases. The challenges lie in recapitulating the cellular heterogeneity, the complexity of the 3D microenvironment, and the related in vivo signaling in 2D culture [6]. Organoid models address many of these unmet requirements of 2D cell models. A 3D in vitro model can reflect the organ-specific functions and morphological characteristics of groups of cells with multi-layered structures, thereby enabling to match essential criteria to construct an in vivo organ. Protocols for generating organoids from hPSCs or adult tissues have been developed for the human brain, liver, lung, and kidney [7]. While tremendous progress has been made in organoid technology, there remain several limitations in the inability to establish an interacting and integrating model that encompasses organoids, vascularized structures, and immune cells for some organs. This necessitates selection of the most suitable model based on the objectives, requirements, and conditions of the research. The optimum approach to recapitulating in vivo complexity is to use a combination of several in vitro models, which can offer a comprehensive and reliable insights of the pathophysiology involved in human diseases. We aimed to recapitulate in vivo physiology by employing various in vitro models to enhance our understanding of human pathophysiology and facilitate the discovery of novel drugs for hereditary hemorrhagic telangiectasis (HHT), a genetic vascular disorder [1, 7, 8].

HHT is an autosomal-dominant disorder characterized by vascular dysplasia. HHT abnormalities, known as telangiectases, are caused by enlarged and dilated capillaries lacking pericytes and smooth muscle cells (SMCs) coverage in the vascular structures, ultimately leading to hemorrhage. Severe defects can lead to fatality via the formation of abnormal vascular structures characterized by direct connections between arteries and veins—known as arteriovenous malformations (AVMs)—in the lungs, brain, liver, and gastrointestinal tract [9, 10]. Most cases of HHT are caused by heterogeneous mutations in the *Endoglin* (*ENG*) and *Activin receptor-like kinase 1* (*ACVRL1*) genes, which are predominantly expressed in vascular ECs [11]. *ENG* and *ACVRL1* are involved in the transforming growth factor-β (TGF-β) and bone morphogenetic protein (BMP) signaling pathways, which regulate vascular patterning [12, 13]. *ENG* encodes an auxiliary coreceptor that enhances TGF-β/BMP signaling by cooperating with ACVRL1 [14, 15]. The lack of appropriate experimental in vitro models has hampered research on the pathogenesis of HHT. Mouse models of HHT harboring the causative genetic aberrations show vascular dysfunction but do not replicate the phenotypes of patients with HHT [16]. The umbilical vein and blood vessels in placental villi of HHT1 groups show significantly reduced endoglin expression but fail to recapitulate pathological signs; they have intact blood vessels and unaltered placental structures [17]. The poor proliferation of ECs derived from peripheral blood monocytes renders them unsuitable for reproducible disease modeling and drug discovery. Thus, the available models do not provide sufficient information on disease-associated defects, implicating requirements of applicable for patients.

The present study aimed to offer more accessible clinical insights into disease predisposition by employing various efficient and scalable in vitro systems. Genome editing-induced haploinsufficiency of *ENG* enabled hPSC-based recapitulation of HHT. The findings showed impaired proliferation, early senescence, and enhanced susceptibility to inflammatory stimuli in ECs but not in SMCs; defective interactions between SMCs and pericytes with ECs; artery-specific impairments; and aberrant downregulation of the Notch pathway. The decreased survival of ECs following inactivation of the Notch pathway suggests a pharmacological intervention strategy for HHT. Overall, we provided optimized research strategies to enhance our understanding of HHT and established valuable insights for pathogenic analysis and the development of novel therapeutic interventions.

## Methods

### Culture of human PSCs

H9 cells (WiCell) were cultured on Matrigel-coated plates in mTeSR1 medium (Stem Cell Tech) at 37 °C under 5% CO_2_. The cells were passaged using ReLeSR (Stem Cell Tech) at a ratio of 1:10–20 with mTeSR1 supplemented with Y-27632 (MedChemExpress). Medium was exchanged for fresh medium daily.

### Reprogramming and genome editing of ENG^+/-^ cells

As a targeting vector, the EF1-mCherry-IRES-Puro-ENG KO donor plasmid containing the left and right homology arms was amplified from hPSC genomic DNA (gDNA) by conventional PCR. The nucleotide sequence of *ENG* was obtained from the NCBI database (Ensembl, ENSG00000106991). The Eng KO episomal CRISPR/Cas9 plasmid contained an *ENG* sgRNA targeting CACCGCACGTGGACAGCATGGACCG.

For genome editing, hPSCs were harvested with Accutase and single cells were resuspended in P3 Primary Cell 4D-Nucleofector Solution (Lonza) with 1.5 µg *ENG* knockout plasmid and 1.5 µg spCas9 *ENG* guide RNA. After electroporation, cells were transferred to the wells of a Matrigel-coated six-well plate containing mTeSR1 with Y-27632. Medium was exchanged daily and 1 µg/mL puromycin was added for selection of purified ENG^+/-^ cells. Single colonies were transferred to one well of a coated 24-well plate and subjected to genotyping, RT-PCR, and Western blotting.

### Differentiation of three germ layers

H9 cells were differentiated using the STEMdiff™ Trilineage Differentiation Kit (Stem Cell Tech) in accordance with the manufacturer’s protocols. Four to six days after single-cell seeding, the cells were fixed and detected using lineage-specific markers for the endoderm (SOX17, FOXA2), mesoderm (TBXT), and ectoderm (Nestin, PAX6).

### Differentiation and purification of ECs

hPSCs were seeded on Matrigel as described previously[18]. Briefly, medium was exchanged for N2B27 medium supplemented with 8 μM CHIR-99021 (Tocris Bioscience) and 25 ng/mL BMP4 (R&D Systems) for lateral mesoderm induction. On day 3, the medium was exchanged for StemPro-34 SFM medium (Gibco) supplemented with 200 ng/mL VEGF-165 (R&D Systems) and 2 μM forskolin (Sigma-Aldrich) for EC induction. On day 6 of differentiation, ECs were harvested using Accutase solution (Sigma-Aldrich) and resuspended in MACS buffer (DPBS supplemented with 0.5% FBS and 2 mM EDTA). The cells were isolated using the CD31 Microbead Kit, Human (Miltenyi Biotec), following the manufacturer’s instructions. After incubation with beads, CD31^+^ cells were immediately isolated using the MidiMACS Separator and Starting Kit (LS) (Miltenyi Biotec) and cultured in EGM-2 Bulletkit Medium (Lonza).

### Differentiation of SMCs

To induce SMC differentiation [19], medium was exchanged for ectoderm induction medium, consisting of E6 medium (Stem Cell Tech) supplemented with 10 μM SB-431532 (Sigma-Aldrich) and 20 ng/mL bFGF (R&D Systems). On day 6, the medium was exchanged for E6 medium supplemented with 10 ng/mL PDGF-BB (PeproTech) and 2 ng/mL TGF-β (PeproTech) until day 12.

### Differentiation of BVOs

To induce BVO differentiation from hPSCs, single cells were aggregated as spin EBs as described previously [20]. In brief, hPSCs were dissociated using Accutase and seeded on ultra-low attachment 96-well plates (Corning) in mTeSR1 medium supplemented with Y-27632. The medium was exchanged for N2B27 medium supplemented with 12 μM CHIR-99021 and 30 ng/mL BMP4. On day 3, the medium was exchanged for N2B27 medium supplemented with 100 ng/mL VEGF-165 and 2 μM forskolin. The next day, the specimens were embedded and the medium was exchanged for StemPro-34 SFM medium supplemented with 100 ng/mL VEGF-165 and 100 ng/mL bFGF until day ≥ 15.

### Differentiation of arteries and veins

To differentiate arteries and veins [5, 25], single cells were seeded on Matrigel-coated plates. Medium was exchanged for CDM2 basal medium with cytokines and chemicals at 24 h intervals: Day 1 for mid primitive streak induction, Day 2 for lateral mesoderm induction, and Days 3–4 for artery or vein induction.

### Quantitative real-time polymerase chain reaction (qRT-PCR)

RNA was extracted from day 6 ECs and day 12 SMCs using the RNeasy Mini Kit (Qiagen) and cDNA was synthesized using ReverTra Ace-α (Toyobo) according to the manufacturer’s instructions. Quantitative PCR analysis was performed using TB Green Premix (TaKaRa) and StepOne software. *GAPDH* was used to normalize the expression levels of target genes and the relative quantification of gene expression was performed using the 2^–ΔΔCt^ method. The sequences of the primers are listed in Supplementary Table S1. Every primer was confirmed by blasts.

### Western blotting analysis

Cells were lysed in protein lysis buffer containing protease inhibitor cocktail (Sigma-Aldrich) and PhosSTOP (Roche). The protein concentration was determined using the Pierce BCA Protein Assay Kit (Thermo) and SpectraMax microplate reader. After blocking using Everyblot buffer (Bio-Rad), primary antibodies against the following factors were added: Endoglin (Abcam, ab169545), HES1 (CST, 11,988), HES5 (Abcam, ab19411), HEY1 (Gene Tex, GTX118007), DLL4 (CST, 2589), p-AKT (CST, 4060), AKT (CST, 9272), p-ERK (CST, 9101), ERK (CST, 4695), p-P38 (CST, 9211), P38 (CST, 9212), CD31 (Thermo, MA5–29474), OCT-4 (CST, 2750), NANOG (Abcam, ab173368), calponin-1 (Santa, sc-58707), α-SMA (Sigma, A2547), and β-actin (Santa Cruz Biotechnology, sc-47778). Next, HRP-conjugated anti-mouse, anti-rabbit, and anti-goat secondary antibodies diluted 1:3000 in Everyblot blocking buffer (Bio-Rad) were added. The membranes were imaged using Clarity Western ECL Substrate (Bio-Rad).

### Flow cytometry

Cells were dissociated in enzyme-free dissociation buffer and filtered in a 5 mL round-bottom polystyrene test tube with a cell strainer snap cap (Falcon). hPSCs were stained with FITC-conjugated anti-OCT-4, anti-NANOG, anti-SSEA-4, and anti-TRA-1–60 antibodies, and ECs were stained with FITC-conjugated anti-CD31 and anti-CD144 antibodies. BVOs were detected using APC-conjugated anti-α-SMA and PDGFR-β antibodies. To detect arteries and veins, anti-SOX17 and anti-NR2F2 antibodies, respectively, were used. Staining using antibodies against surface and nuclear factors was performed using DPBS and 1 × perm/wash, respectively. Cell populations were analyzed using an FACSAria instrument with FACS Diva software (BD Biosciences).

### Immunocytochemistry (ICC)

Cells were fixed with fixation and permeabilization solution (BD Biosciences) for 15 min and blocked in 1 × perm/wash buffer (BD Biosciences) for 30 min. Then they were immediately reacted with unconjugated primary antibodies against SOX-17, FOXA2, TBXT, NESTIN, PAX-6, MIXL1, PECAM-1, VE-CADHERIN, CALPONIN-1, SM22α, and α-SMA as well as with Alexa 488-conjugated anti-SSEA-4, anti-OCT-4, anti-SOX-2, and anti-ki-67 antibodies. The Alexa 488-conjugated secondary antibodies targeted the host species of the corresponding unconjugated primary antibodies. Nuclei were stained with Hoechst 33342 (Invitrogen).

### Tubule formation assay

Tube formation by ECs was analyzed using a Matrigel assay. Each well of a 24-well tissue culture plate was covered with 300 µL Matrigel and incubated at room temperature for 1 h to solidify the Matrigel. ECs were harvested using Accutase and 2 × 10^5^ were resuspended in 200 µL EGM-2 Bulletkit medium. The cells were transferred to solidified Matrigel and capillary-like structures were visualized after 6 h using a light microscope.

### TUNEL assay

The apoptosis of ECs was analyzed using the in situ Cell Death Detection Kit (Roche) according to the manufacturer’s instructions. ECs were fixed and permeabilized in fixation and permeabilization solution for 1 h at room temperature. Next, they were incubated in TUNEL reaction mixture for 1 h at 37 °C and nuclei were stained with Hoechst 33342.

### Visualization of 5-ethynyl-2-deoxyuridine (EdU)

Cell proliferation was analyzed using the EdU Cell Proliferation Kit (Invitrogen), which detects only cells in the S-phase, according to the manufacturer’s instructions. Briefly, EdU labeling solution (1:500 dilution) was incubated at 37 °C for ≤ 8 h. Cells were imaged using a fluorescence microscope.

### Cytotoxicity assay

The cytotoxicity of TNF-α and PM against ECs was determined using the EZ-Cytox Kit (Dogen) according to the manufacturer’s instructions. Cells were analyzed at a wavelength of 450 nm using a microplate reader.

### Statistical analysis

Experiments were performed in triplicate; results are means ± SEMs. Statistical analysis was performed using GraphPad Prism and Microsoft Excel software. Values of *p* < 0.05 were considered indicative of statistical significance.

## Results

### HHT-hPSCs with *ENG* haploinsufficiency are pluripotent

**Fig. 1:**
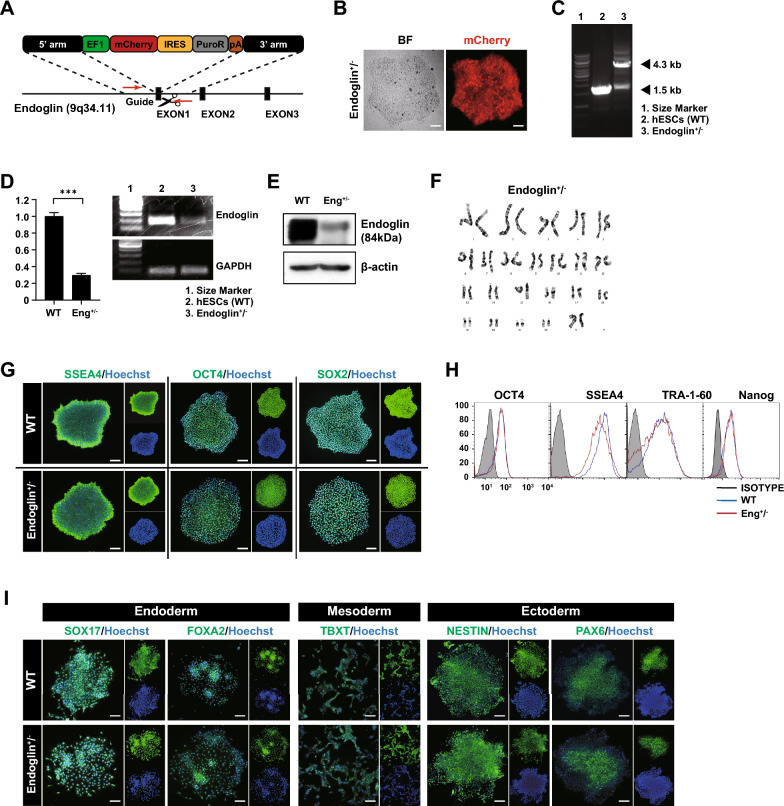
Establishment of *ENG*-haploinsufficient hPSCs. **A **Donor plasmid for gene editing of ENG^+/-^ cells expressing mCherry and PuroR. **B** Expression of mCherry in ENG^+/-^ cells. **C** Genotyping of ENG^+/-^ hPSCs by conventional PCR. **D** Expression of *ENG* at the transcriptional and **E** translational levels in ENG^+/-^ cells, indicating a reduced level of *ENG*. **F** Karyotype analysis of ENG^+/-^ cells by GC binding. **G** Characterization of pluripotent markers by immunocytochemical analysis and **H** flow cytometry. **I** Immunocytochemical analysis of markers of the endoderm (SOX17 and FOXA2), mesoderm (TBXT), and ectoderm (NESTIN and PAX6). Scale bar, 100 µm. (***p < 0.001). Data are the mean value ± SEM. *P*value < 0.05 are depicted and were obtained using two-tailed *t*-test

Given that *ENG* haploinsufficiency leads to the deficiencies observed in patients with HHT [22], and that it is difficult to maintain pluripotency in ENG^−/−^ hPSCs (Fig. S1), we established a stable ENG^**+/-**^ hPSC line by precisely targeting the first exon of the *Endoglin* gene for CRISPR/Cas9-mediated replacement; mCherry was used to visualize target cells (Fig. 1A, B). Accurate integration of the plasmid into the *Endoglin* locus in a single chromosome of the selected hPSC-HHT clone was confirmed by PCR analysis of genomic DNA (Fig. 1C). Analysis of *Endoglin* expression at the transcriptional and translational levels revealed haploinsufficiency in *ENG* expression, a hallmark of HHT (Fig. 1D, E). ENG^**+/-**^ cells showed a normal female karyotype (46, XX); no chromosomal modification occurred during *ENG* knockout (Fig. 1F). The hPSC-HHT clone expressed pluripotency markers (*e*.*g*., OCT4, SOX2, Nanog, SSEA4, and TRA-1–60), and was capable of differentiation into three germ cell lineages (Fig. 1G–I), indicating normal pluripotency despite insufficient endoglin expression.

### ECs differentiated from HHT-hPSCs display abnormal defects

**Fig. 2:**
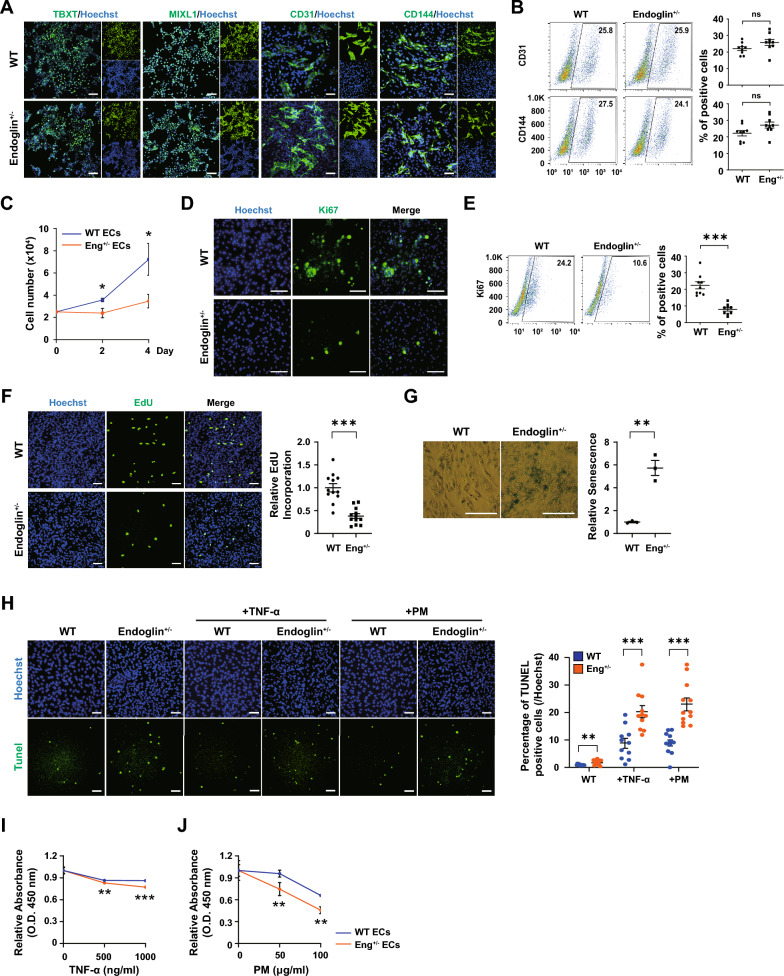
Differentiation, proliferation, and cytotoxicity of WT vs ENG^+/-^ ECs. **A** Immunocytochemical analysis of ECs derived from WT and ENG^+/-^ cells in terms of the expression of TBXT, MIXL1, CD31, and CD144. **B** Flow cytometry of the EC markers, CD31 and CD144. (n = 9) **C** Growth of CD31^+^ ECs at days 0, 2, and 4 after isolation by MACS. **D** Immunostaining and **E** flow cytometry of the cell cycle marker Ki67 (green) with Hoechst 33342 counterstaining (blue). (n = 9) **F** Immunostaining for EdU, a marker of the S-phase (green), with Hoechst 33342 counterstaining (blue). (n = 12) **G** Senescence assay of ECs and WT and ENG^+/-^ ECs. (n = 3) **H** TUNEL assay of WT ECs, and ENG^+/-^ ECs treated without or with TNF-α and PM2.5. (n = 11–12) **I** Cytotoxicity assay of the effects of treatment with TNF-α (0, 500, and 1000 ng/mL) and **J** PM (0, 50, and 100 μg/mL). (n = 4–5) Scale bars, 100, 200 µm. (*p < 0.05, **p < 0.01, and ***p < 0.001). Data are the mean value ± SEM. *P*value < 0.05 are depicted and were obtained using two-tailed *t*-test

To gain pathophysiological insights into HHT patients, we first conducted direct differentiation of HHT-hPSCs into ECs, one of the major vascular cell types, using a previously established method outlined schematically in Fig. S2A [18]. ECs derived from HHT-hPSCs exhibited comparable morphological features to controls (Fig. S2B). WT and ENG^+/-^ cells displayed similar gene expression patterns over the 7-day differentiation period, characterized by downregulation of pluripotency-related genes (*OCT4* and *NANOG*), transient upregulation of an early mesodermal gene (*TBXT*), and upregulation of mesodermal (*ISL1*) and endothelial (*VE-CADHERIN*, *PECAM-1*, and *KDR*) markers (Fig. S2C). These changes were reflected in the protein levels determined by ICC and immunoblotting (Figs. 2A and S2D). The populations of differentiated ECs were similar for both types of hPSCs, indicating comparable differentiation efficacy (CD31: WT = 25.8%, ENG^+/-^= 25.9% & CD144: WT = 27.5%, ENG^+/-^ = 24.1%) (Fig. 2B).

The pure population of ECs (isolated using an anti-PECAM-1 antibody) derived from both hPSCs exhibited a similar ability to form tube-like structures on Matrigel, indicating the presence of functional ECs (Fig. S2E, S2F). However, ECs derived from HHT-hPSCs lost their proliferation ability, as indicated by suppressed growth and Ki67 expression, a marker of proliferation (Ki67: WT = 24.2%, ENG^+/-^ = 10.6%) (Fig. 2C-E). DNA replication, monitored by measuring EdU incorporation, indicated reduced proliferation of ECs derived from HHT-hPSCs compared to ECs from normal hPSCs (ENG^+/-^ EC EdU relative to WT = 0.31 folds) (Fig. 2F). Instead, these ECs underwent senescence, as demonstrated by SA-β-gal staining, an established hallmark of cellular senescence. Quantified levels of SA-β-gal staining showed increased the frequency of positive cells under downregulation of *Endoglin* expression (ENG^+/-^ EC SA-β-gal relative to WT = 5.73 folds) (Fig. 2G). Considering their propensity for senescence, which may lead to diminished viability, we assessed HHT-EC apoptosis using a TUNEL assay. Notably, under basal conditions, HHT-ECs showed a higher prevalence of TUNEL positivity compared to control ECs (Fig. 2H). Moreover, when subjected to apoptotic stimuli from TNF-α, an inflammatory cytokine recognized for its capacity to induce pathology in ECs, HHT-ECs resulted in an increased TUNEL-positivity rate and enhanced cytotoxicity. These findings strongly implicate that ECs with *Endoglin* haploinsufficiency were more sensitive to such stressors (Fig. 2H, I). The effect of particulate matter of < 2.5 µm aerodynamic diameter (PM2.5), an air pollutant that promotes inflammation and endothelial dysfunction [23], on patients with HHT is unknown. Exposure of HHT-ECs to PM2.5 resulted in a substantial dose-dependent increase in cytotoxicity, confirming the heightened susceptibility of HHT-ECs to adverse environmental conditions (Fig. 2H, J).

### SMCs derived from HHT-hPSCs exhibit no discernible abnormalities

**Fig. 3:**
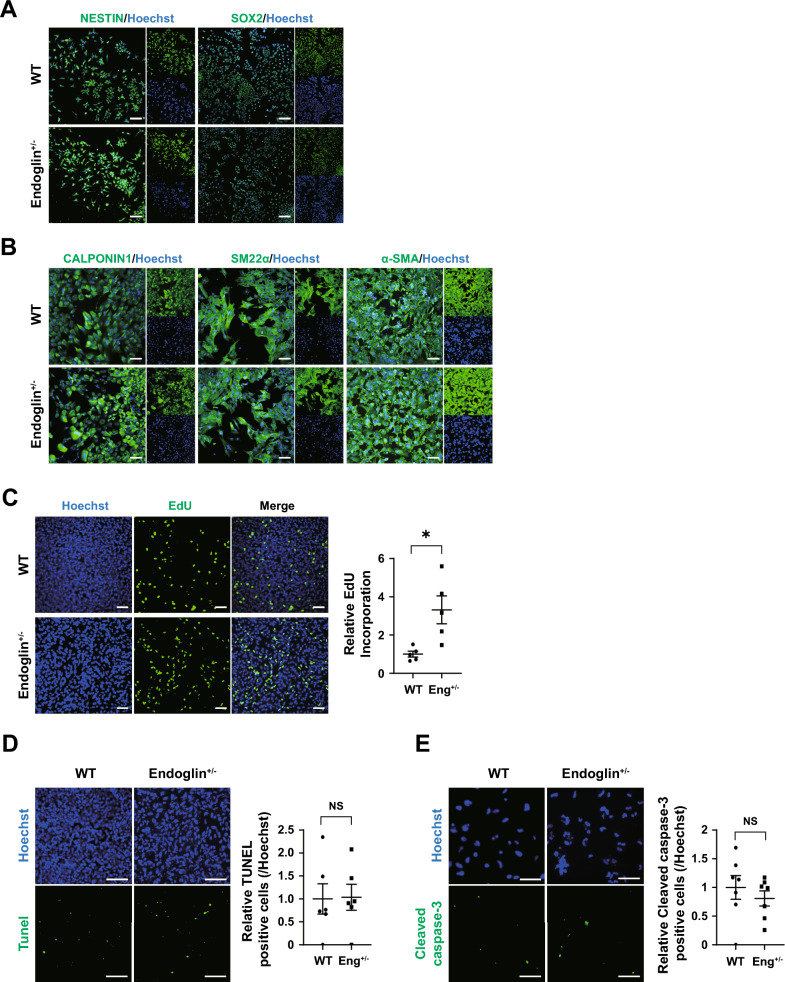
Differentiation of SMCs from hPSCs. **A** Immunocytochemical analysis of ectodermal (NESTIN and SOX2) and **B** smooth muscle (CALPONIN1, SM22-α, and α-SMA) markers with Hoechst 33342 nuclear counterstaining. **C** Immunostaining for EdU, a marker of the S-phase (green). (n = 5) **D** Apoptosis of SMCs as determined by TUNEL assay. (n = 6) **E** Immunocytochemical analysis of cleaved caspase-3 expression in WT and ENG^+/-^ SMCs; there was no significant difference. (n = 7) Scale bars, 100, 200 µm. (*p < 0.05). Data are the mean value ± SEM. *P*value < 0.05 are depicted and were obtained using two-tailed *t*-test

Based on the central function of SMCs in vascular tissues and our profound interest in exploring their potential implications for HHT patients, we triggered direct differentiation of HHT-hPSCs into SMCs (Fig. S3A) [19]. During the neuroectoderm-intermediate differentiation phase, WT and ENG^+/-^ cells displayed similar gene expression patterns, which were characterized by downregulation of pluripotency-related genes (*OCT4* and *NANOG*), followed by upregulation of neural stem cell-related genes (*SOX2*, *NESTIN*) and subsequently significant upregulation of SMC markers (*CNN1*, *SM22-α*, and *α-SMA*) (Fig. S3B). These morphological and genetic changes were supported by the SMC-like morphology of the cells and by the results of immunocytochemical and immunoblotting analyses of the levels of the encoded proteins (Figs. 3A, B, S3C). However, no significant differences in the expression of differentiation markers were observed between the control and HHT-hPSCs during SMC differentiation. To investigate whether SMCs lost their proliferation ability (similar to ECs derived from HHT-hPSCs), we assessed EdU incorporation; SMCs displayed more active EdU incorporation than ECs, indicating an enhanced cell proliferation capability (ENG^+/-^ SMC EdU relative to WT = 3.32 folds) (Fig. 3C). Furthermore, under basal conditions, TUNEL analysis and evaluation of cleaved caspase-3 expression indicated small numbers of apoptotic HHT and control SMCs (Fig. 3D, E). Taken together, these results confirm EC-specific impairment in patients with HHT and that ECs are highly sensitive to apoptotic insults. Therefore, through direct differentiation into specific blood vessel cell types, we have identified the cell types affected by the disease and elucidated their distinctive characteristics.

### Three-dimensional vascular organoids derived from HHT-hPSCs reflect aberrant vascular organization

**Fig. 4:**
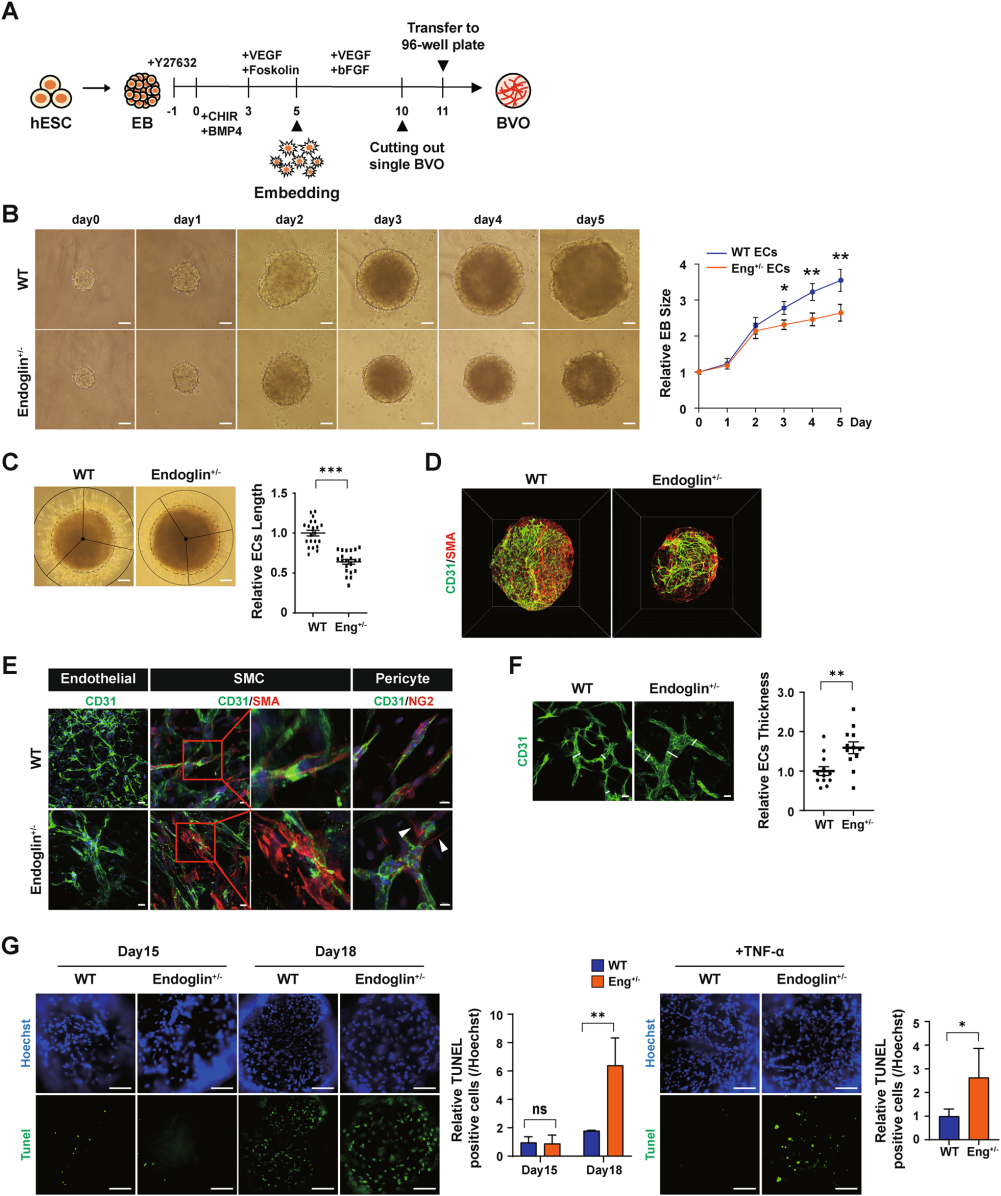
Differentiation of blood vessel organoids from hPSCs. **A** Protocol for differentiation of blood vessel organoids. **B** Bright-field images and growth curves on days 0–5 of differentiation. (n = 10) **C** Length of ECs after embedding compared to WT- and ENG^+/-^-BVOs. (n = 21) **D** Three-dimensional structure of BVOs by Lightsheet, CD31, and SMA. **E** Immunocytochemical analysis of markers of ECs (CD31), SMCs (SMA) and pericytes (NG2) in BVOs. F Thickness of endothelial cells compared to WT and ENG^+/-^ BVOs. (n = 12) **G** Representative images of TUNEL staining of apoptosis in BVOs at days 15 and 18 and after TNF-α treatment. Scale bars, 100, 200 µm. (n = 4–6) (*p < 0.05, **p < 0.01, and ***p < 0.001). Data are the mean value ± SEM. *P*value < 0.05 are depicted and were obtained using two-tailed *t*-test

Organoids recapitulate the 3D milieu in tissues or organs, including normal and pathological development, intercellular communication, and tissue- or organ-specific functions in vitro. The robust development of blood vessel organoids (BVOs), including ECs and mural cells (MCs), which promote self-assembly of capillary networks with a basement membrane, provides a potent platform for investigating the genetic vascular disorder of HHT. Furthermore, these organoids enable the investigation of EC–MC crosstalk and the identification of structural defects. We evaluated the ability of HHT-hPSCs to form 3D vascular structures (Fig. 4A) [20]. By contrast to the control, early BVO aggregates with HHT features, induced to adopt a mesodermal fate, showed compromised growth dynamics, despite an identical initial seeding density (Fig. 4B). In addition, HHT-BVOs showed significantly reduced sprouting vasculature in a 3D collagen I-Matrigel matrix (ENG^+/-^ BVO length relative to WT = 0.64 folds) (Fig. 4C). By day 15, BVOs derived from WT and ENG^+/-^ cells exhibited vascular structures positive for EC and MC markers (Fig. 4D). However, the ratios of ECs to SMCs and pericytes were reduced in HHT-vascular organoids (Fig. S4A). Immunostaining using antibodies against CD31/SMA and CD31/NG2 revealed reduced coverage of MCs on HHT-ECs, and MCs were positioned more distally from ECs compared to WT-ECs (Fig. 4E). In addition, there was a significant increase in the thickness of the CD31-positive EC layer in HHT-BVO (ENG^+/-^ BVO thickness relative to WT = 1.60 folds) (Fig. 4F), suggesting the presence of defective microvascular structures in the context of *ENG* haploinsufficiency. Considering the propensity of HHT-ECs towards diminished cell viability even under basal conditions, we assessed cell viability in the 3D vascular structures via TUNEL analysis. TUNEL-positive cells were rare in HHT- and control-BVOs at day 15, but their frequency was significantly higher in HHT-BVOs up to day 18. Furthermore, they exhibited heightened sensitivity to TNF-α, as did directly differentiated ECs (Fig. 4G). Therefore, the 3D system provides clear evidence that HHT involves intrinsic defects of vascular structure and unstable sensitivity to apoptotic insults. Moreover, with direct differentiation into ECs and SMCs, it becomes more evident that these intrinsic defects are more pronounced in ECs than in SMCs.

### Distinct effects of *ENG* mutation in arteries and veins

**Fig. 5:**
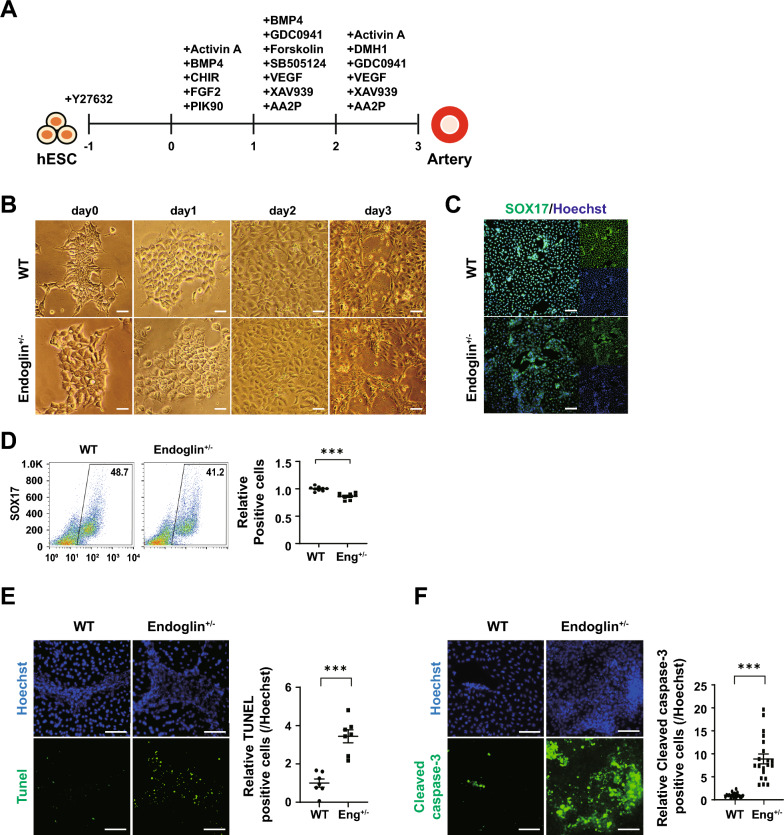
Generation of arteries from hPSCs. **A** Artery generation protocol. **B** Bright-field images obtained on days 0–3 of artery generation. **C** Immunocytochemical analysis of an arterial marker (SOX17) with Hoechst 33342 counterstaining. **D** Flow cytometry for SOX17 at day 3 of artery differentiation. (n = 9) **E** TUNEL assay of apoptosis at day 3. (n = 7) **F** Immunocytochemical analysis of cleaved caspase-3 in WT and ENG^+/-^ arteries. (n = 20) Scale bars, 100, 200 µm. (***p < 0.001). Data are the mean value ± SEM. *P*value < 0.05 are depicted and were obtained using two-tailed *t*-test

Mouse models have shown that ECs with *ENG* mutations produce venous rather than arterial features, together with secondary proliferation and expansion, leading to the development of AVM [24]. We established an in vitro system that allowed differentiation into arterial and venous EC subtypes to assess the similarity of the resulting arterial malformations to those observed in vivo. To investigate the effect of *ENG* mutation on the behaviors of the EC subtypes, we employed cutting-edge differentiation methods to generate well-defined populations of arterial and venous ECs [5]. Arterial ECs, generated by lateral mesodermal differentiation, had morphological features comparable to the control and expressed the artery marker SOX17 (Fig. 5A–C). However, the population of differentiated arterial ECs was slightly suppressed in HHT-hPSCs, suggesting impaired differentiation into the arterial EC subtype (WT = 48.7%, ENG^+/-^ = 41.2%) (Fig. 5D). Considering the predisposition of HHT-ECs toward decreased viability, we assessed endogenous cell stability by performing a TUNEL assay and analyzing the expression of cleaved caspase-3. HHT-arterial ECs showed higher rates of TUNEL positivity and cleaved caspase-3 positivity (Fig. 5E, F).

**Fig. 6:**
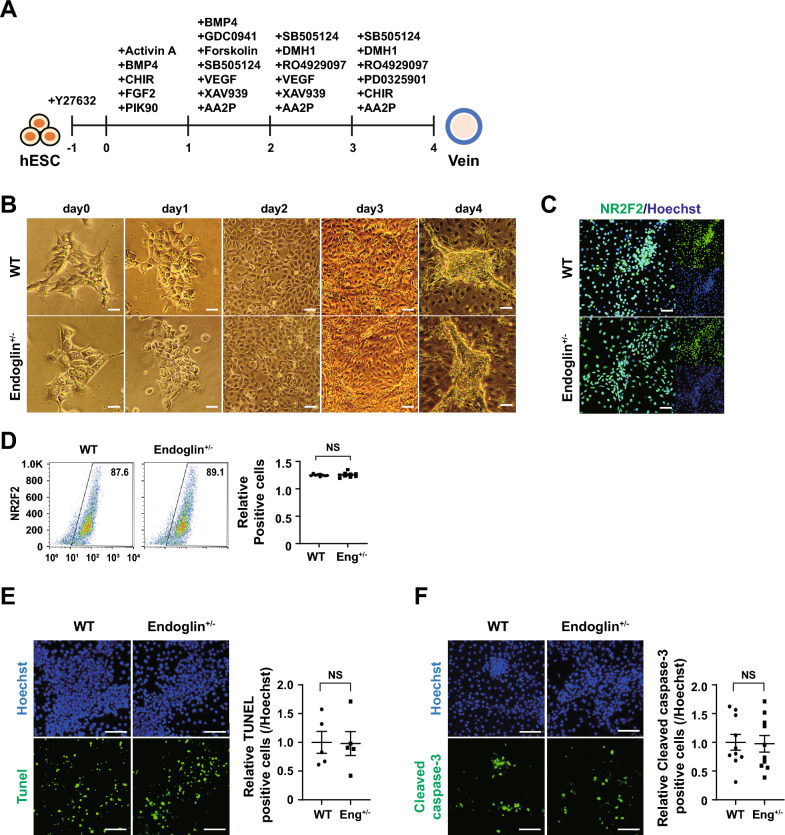
Generation of veins from hPSCs. Vein generation protocol. **B** Bright-field images on days 0–4 of vein generation. **C** Immunocytochemical analysis of a venous marker (NR2F2) with Hoechst 33342 counterstaining. **D** Flow cytometry of NR2F2 at day 4 of vein differentiation. (n = 9) **E** TUNEL assay of apoptosis at day 4. (n = 5) **F** Immunocytochemical analysis of cleaved caspase-3 in WT and ENG^+/-^ veins. (n = 10) Scale bars, 100, 200 µm. Data are the mean value ± SEM. *P*value < 0.05 are depicted and were obtained using two-tailed *t*-test

Next, we investigated whether the EC features of HHTs were also persisted in veins by generating well-defined venous populations (Fig. 6A). hPSCs from WT and ENG^+/-^ sources differentiated into venous ECs, which exhibited congruent morphological features throughout differentiation. Immunocytochemical analysis confirmed the expression of the vein marker NR2F2 in both populations (Fig. 6B, C). Compared to arterial ECs, ENG^+/-^ hPSCs did not display any specific propensity for differentiation potential, showing similar differentiation into vein ECs as control (WT = 87.6%, ENG^+/-^ = 89.1%) (Fig. 6D). Furthermore, the predisposition of HHT-artery into impaired cell viability did not persist in vein ECs. The numbers of TUNEL and cleaved caspase-3-positive cells in venous ECs were comparable to those of the control (Fig. 6E, F). We additionally confirmed other clone, ENG^+/-^ clone 2, by differentiating ECs, SMCs, and artery, which indicated similar results with HHT-hPSCs (Fig. S5). Therefore, the EC subtype model underscore more distinct features of *ENG* haploinsufficiency, which is not able to be delineated from 2D endothelial differentiation and 3D organoid models. This highlights the importance of employing multiple in vitro systems when investigating disease pathophysiology.

### Function of Notch signaling in impaired EC stability in HHT

**Fig. 7:**
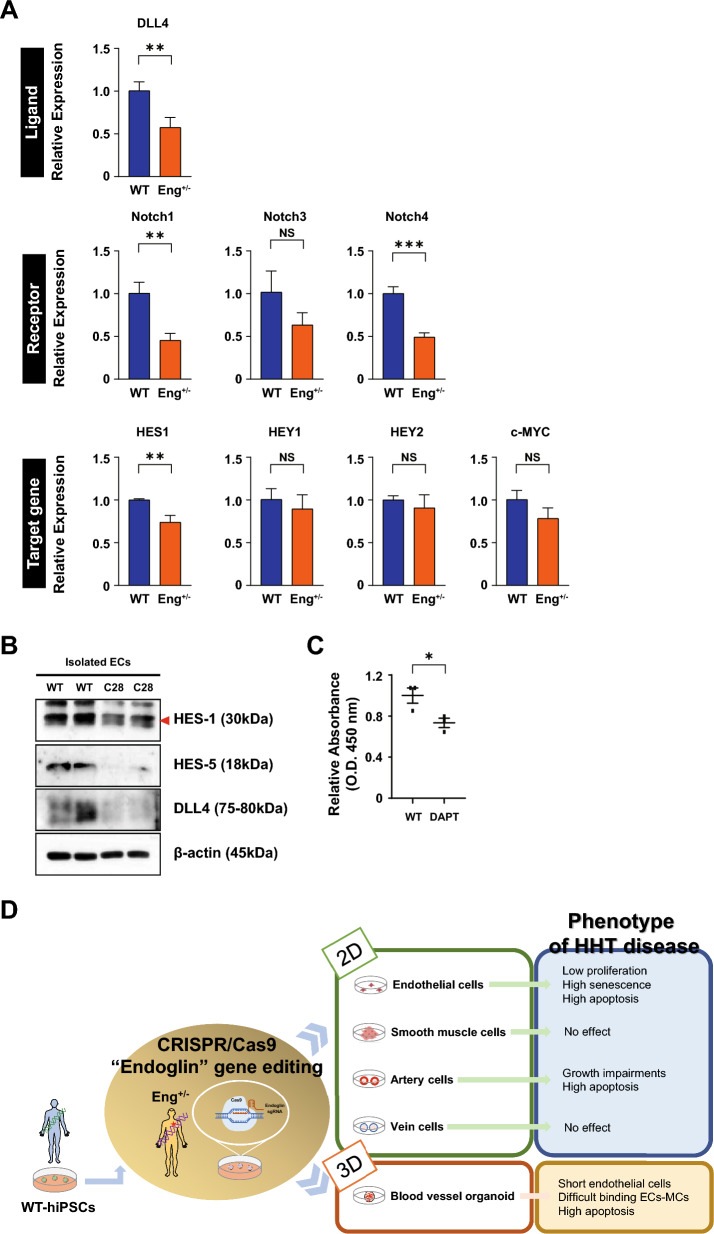
Notch signaling in ECs. **A** qRT-PCR analysis of a ligand (DLL4), receptor (Notch 1, 3, and 4), and target (*HES1*, *HES5*, *HEY1*, *HEY2*, and *c-MYC*) genes. (n = 3–4) **B** Western blotting analysis of Notch signaling-related factors. **C** Cytotoxicity assay of WT-ECs treated with DAPT. (n = 3) **D** Graphic conclusions of diverse pathogenesis in HHT-hPSCs. (*p < 0.05, **p < 0.01, and ***p < 0.001). Data are the mean value ± SEM. *P*value < 0.05 are depicted and were obtained using two-tailed *t*-test

Given the versatility of in vitro modeling, which provides amenable systems for disease modeling and allowing the exploration of regulatory pathways and factors, we next evaluated the molecular mechanisms underlying the detrimental phenotypes of ECs derived from HHT-hPSCs, such as growth arrest and senescence. To investigate the signaling pathways involved, we conducted immunoblotting for components of the ERK, p38 MAPK, and AKT pathways. However, there was no significant change in kinase phosphorylation in HHT-ECs compared to controls (Fig. S4A). Notch signaling is implicated in angiogenesis, tip/stalk EC specification, and the expression of arterial markers, including modulation of VEGF signaling [25, 26]. Furthermore, the regulation of Notch signaling in ECs is required for proper vascular morphogenesis and may have implications for the pathophysiology of AVMs [27, 28]. The expression of the Notch ligand DLL4 was markedly suppressed in ECs with the *ENG* mutation. In addition, Notch receptors Notch 1 and 4, but not 3, were significantly downregulated, resulting in the suppression of target genes at the transcriptional level (Fig. 7A). Western blotting corroborated the downregulation of Notch signaling-related proteins in ECs with HHT features (Fig. 7B). To assess the correlation between the downregulation of Notch signaling in ENG^+/-^ cells with the heightened vulnerability of ECs, we used DAPT to downregulate Notch signaling in normal ECs; the result was significantly reduced viability (Fig. 7C). These results suggest that autonomous downregulation of Notch may indeed be associated with the features of HHT-ECs.

## Discussion

Our findings indicate the importance of employing multiple scalable in vitro systems, encompassing genetic modification and differentiation of hPSCs, to enhance our understanding of disease pathogenesis. Two-dimensional cellular models are preferable to animal or tissue models due to their rapidity, cost-effectiveness, and controllability. Despite these advantages, cellular models do not accurately replicate human physiology, such as developmental processes and interactions between cells and tissues [29, 30]. Nevertheless, insights from 2D models are likely to elucidate the direct effect of ENG protein haploinsufficiency on cells that organize blood vessels, such as ECs and SMCs, without the compensatory mechanism typical of in vivo disease models [31]. HHT-ECs showed reduced proliferation, early senescence, abnormal stability, and heightened sensitivity to toxic insults. These observations align with earlier studies that employed knockdown models using small interfering RNA (siRNA) in mouse embryonic ECs, which showed reduced EC proliferation and TGF-β signaling [14]. However, in a 2D model derived from HHT^c.1678>T^-hiPSCs, ENG haploinsufficiency had no effect on EC function in terms of proliferation, barrier function and sprouting angiogenesis compared to isogenic controls [32]. However, under microfluidic flow, HHT-specific defective vascular organization was observed, accompanied by clear evidence of vascular leakage. Although haploinsufficiency is accepted as the primary mechanism underlying HHT1, it fails to account fully for the variability in clinical manifestations inter- and intra-families, nor does it explain the selective impact on specific vascular beds despite the ubiquitous presence of mutated genes across the vasculature [33, 34]. There is reportedly a critical threshold of endoglin expression, below which ECs exhibit inadequate responses to environmental stimuli and become highly susceptible to HHT1 mutation. Additionally, somatic mutations resulting in a second (bi-allelic) genetic hit in ENG/Alk1 within vascular lesions from HHT patients are important, consistent with the requirement for bi-allelic loss of function, to reproduce AVM formation in animal models [35]. Thus, the distinct expression patterns of endoglin in hPSCs, whether from genetically engineered haploinsufficient models or patient samples, may explain the disparities in results from 2D culture. The expression level of ENG should be considered in in vitro modeling.

A recent study involving conditional endoglin expression in endothelial and vascular SMCs facilitated a cell type-specific analysis of endoglin’s effects. In-depth examination of the effect of endoglin on in vivo SMCs showed that SM22α-driven conditional endoglin expression increased the thickness of the vascular SMC layer [36]. Additionally, loss of endoglin in Pax3-positive cells enhanced smooth muscle actin expression and SMC disorganization [37]. Nevertheless, the intricate regulation of SMC-specific gene expression, particularly in the context of vessel formation, hampers investigation of abnormal endoglin expression and its effect on a variety of cell types using in vivo systems. Our SMC 2D models with ENG^±^ expression showed that dysregulation of endoglin expression did not cause discernable abnormalities of SMCs (Fig. 3). The proper assembly of the vessel wall relies on reciprocal communication between ECs and SMCs involving endoglin and ALK-1 for SMC recruitment or differentiation in the developing vasculature [38, 39]. Endothelial endoglin mediates interactions between ECs and VSMCs by binding to integrin, likely integrin α5β1, facilitating the recognition of ECM proteins expressed by vascular SMCs and promoting adhesion and recruitment to blood vessels [40]. Thus, our 2D model allows for the assessment of direct effects in individual cells comprising the vasculature as a result of ENG mutation. However, understanding of the collective outcome influenced by interactions in the vasculature environment and subsequent aberrant SMCs, hallmark traits associated with HHT, is limited.

The application of 3D blood vessel organoids has expanded our ability to explore the microvasculature in patients with HHT, enabling comprehensive examination of its morphological, functional, and molecular aspects. A 3D model enabled the detection of multiple structural defects in ENG^+/-^ mutant mice, including compromised angiogenic sprouting vasculature, reduced MC coverage on ECs (positioned distally), and the emergence of enlarged EC structures. Furthermore, the 3D system revealed inherent defects in the viability of vascular structures and heightened sensitivity to inflammatory factors (Fig. 4H). Moreover, targeted differentiation into arterial and venous subtypes has deepened our understanding of the effect of HHT on vessel subtypes—impaired arterial differentiation accompanied by increased cell-autonomous damage in arteries, but not in veins (Fig. 5). Loss-of-function mutations in *ENG*, primarily found in ECs, have been associated with the acquisition of arterial rather than venous features, contributing to vessel expansion and AVM development, with arterioles implicated as the primary sites of malformation [24]. The reduced expression of arterial markers, like Ephnb2, together with venous markers (*e*.*g*., ephrin B4 and apelin receptor), in the absence of *ENG* expression suggests the presence of arterial defects [41]. However, the selective loss of ENG expression in veins and capillaries resulted in retinal AVMs, revealing that arterial endoglin failed to protect against AVMs [42]. It is important to note that, although a targeted subtype 2D model allows evaluation of endoglin’s cell-autonomous effects, the absence of microenvironments and communication between arteries, capillaries, and veins hampers analysis of the in vivo mechanisms, which lies beyond the scope of this study.

Advancing disease modeling requires consideration of additional factors contributing to AVM formation, such as somatic mutations, inflammation, pro-angiogenic triggers, and blood flow shear stress [43]. Although many mutated genes in HHT encode components of the TGF-β signaling pathway (*e*.*g*., ENG, ALK1, SMAD4, and GDF2), a recent study identified other genes (INHA, HIFA, DNM2, POSTN, and ANGPTL4) as potential drivers of HHT [44]. Dysregulation of EC responsiveness to hypoxia as a result of mutations in these genes, together with environmental hypoxia, may contribute to vascular abnormalities. The understanding of disease initiation mechanisms varies greatly depending on gene expression patterns. To unravel these complexities, our protocol, which involves genetic derivation into multiple in vitro models, will enable investigation of the correlation between mutations in these newly suggested genes, the hypoxia response, and HHT development. Loss of ENG function leads to abnormal EC responses to VEGF signaling, a key angiogenic factor. This aberrant response to angiogenic stimuli, either developmental or pathological, can contribute to AVM formation when combined with endoglin depletion [43, 45]. ENG also drives the redistribution of ECs by activating migration against the direction of blood flow, and defects in ENG functions lead to aberrant EC shapes, migration, and sizes, resulting in vessel dilation and higher hemodynamic forces, which support AVM enlargement [46, 47]. Our model is focused on ENG defects without these additional drivers inducing ENG loss of function, including blood flow and angiogenic stimuli. Therefore, recent applications of in vitro flow, which induce substantial vascularization and morphological maturation of tissue organoids [48], will enable the recapitulation of AVMs usually enhanced under hemodynamic forces.

Patients with HHT have immune dysregulation, characterized by reduced lymphocyte counts and qualitative abnormalities in neutrophils, resulting in diminished release of neutrophil extracellular traps (NETs) and disrupted cell adhesion and migration [49]. These findings suggest a link between chronic iron deficiency and altered actin cytoskeleton, which may affect immune function. However, roles for genetic aberrations in immune cell specification and neutrophil activation cannot be ruled out. Additionally, severe right heart dysfunction and high output heart failure (HOHF) are rare complications of HHT [50]. Therapeutic options for these complications are limited because of insufficient information and clinical trial data. Furthermore, chronic or complicated symptoms, including impaired immune function and heart failure, have not been replicated in mouse models of HHT. Understanding these multifaceted phenomena may necessitate the integration of several in vitro models. Notably, the use of hPSCs enables the engineering of HHT-inducible genes and differentiation into specific functional cell types, such as those of hematopoietic or cardiac lineages. Recent advances, including the integration of microfluidic technologies with vascularized tissue organoids [51], hold promise for investigating the effect of vascular dysfunction on interconnected organs—such as the pulmonary or cardiac system—in patients with HHT. It is also important to take into account the heterogeneity of hPSCs, as the growing diversity of new PSCs lines can inevitably impact the reproducibility of PSCs-based disease modeling. Variations between donors, genetic stability, and experimental conditions contribute to differences in models by affecting factors such as differentiation efficiency, cellular heterogeneity, and transcriptomic and proteomic profiles [52, 53]. To accurately interpret the results obtained from disease modeling derived from hPSCs, it is essential to consider this heterogeneity, and conducting robust statistical analysis using various hPSC lines is required to obtain reproducible and meaningful outcomes.

Notch signaling is important in vascular morphogenesis, and its dysregulation has been linked to the development of angiogenic defects and AVMs due to loss-of-function mutations [27, 28]. In *ENG*-deficient cells, there is a reduction in the expression of Notch target genes, which can be restored by ALK1 activation, confirming ALK1-dependent regulation of Notch signaling [54]. Acvrl1^–/–^ mouse models have shown that AVM development is associated with the downregulation of Notch1 and Jag1 [55]. Our HHT-EC models also exhibited downregulation of the Notch ligand DLL4 and receptors Notch 1 and 4, supporting a role for Notch in the *ENG*-mediated EC stability. Furthermore, sustained inhibition of Notch signaling induced cell instability, implicating Notch signaling in the autonomous features of ECs in HHT. The interplay between endothelial BMP and SMAD signaling with Notch signaling during vascular development has been documented [56, 57]. Notably, direct interactions between components of signaling pathways, such as the binding of activated SMAD and NICD, lead to the potentiation of HEY1 expression [57]. However, others have failed to detect NOTCH signaling impairment following ENG signaling disruption [58]. Although our findings support a role for Notch in 2D EC models, insights from 3D models, which include a variety of cell types, are crucial for identifying underlying mechanisms. In particular, advancements in single-cell RNA sequencing may provide insight into the correlation between vascular cell organization in the 3D structure and cell-specific effects, as well as related signaling induced by ENG mutations.

Although our model may not fully replicate the phenotype seen in patients with HHT, we consider our combination of models to be an invaluable tool for investigating the underlying mechanisms, especially regarding the EC-mural interaction. Our goal was to use the optimum tools to identify drugs capable of reversing these phenomena and promoting vascular normalization. Incorporating pro-inflammatory triggers, such as inflammatory macrophages, into the model can lead to a more sophisticated representation of disease phenotype. Next-generation models based on our method will contribute to screening for novel therapeutic interventions and drug discovery.

## Conclusions

Overall, we highlight the use of diverse in vitro systems to assess HHT disease through genetic mutations of ENG in hPSC-based recapitulation. Our results reveal impairments in HHT disease, particularly in endothelial cells, with specific deficits in arteries, and downregulation of the Notch pathway. Notably, we implemented a model closely resembling clinical phenomena by applying not only 2D in vitro systems but also 3D blood vessel organoids. The inclusion of various aspects of the disease in our study contributes to a better understanding and provides insights for the personalized medicine.

### Supplementary Information

Supplementary material 1.

Supplementary material 2.

## Data Availability

The datasets used and/or analyzed during the current study are available from the corresponding author on reasonable request.

## Abbreviations

iPSC: Induced pluripotent stem cell
EC: Endothelial cell
ECM: Extracellular matrix
HHT: Hereditary hemorrhagic telangiectasis
SMC: Smooth muscle cell
AVM: Arteriovenous malformation
ENG: Endoglin
ACVRL1: Activin Receptor-like kinase 1
TGF-β: Transforming growth factor- β
BMP: Bone morphogenetic protein
BVO: Blood vessel organoid
MC: Mural cell
