# Supplementary material for: Employment of diverse in vitro systems for analyzing multiple aspects of disease, hereditary hemorrhagic telangiectasia (HHT)
Source: Cell Biosci. 2024 May 22;14:65. doi: 10.1186/s13578-024-01247-z (PMC11110195; doi:10.1186/s13578-024-01247-z)
Supplement: Supplementary file 1 — Supplementary material 1. [file 13578_2024_1247_MOESM1_ESM.docx]

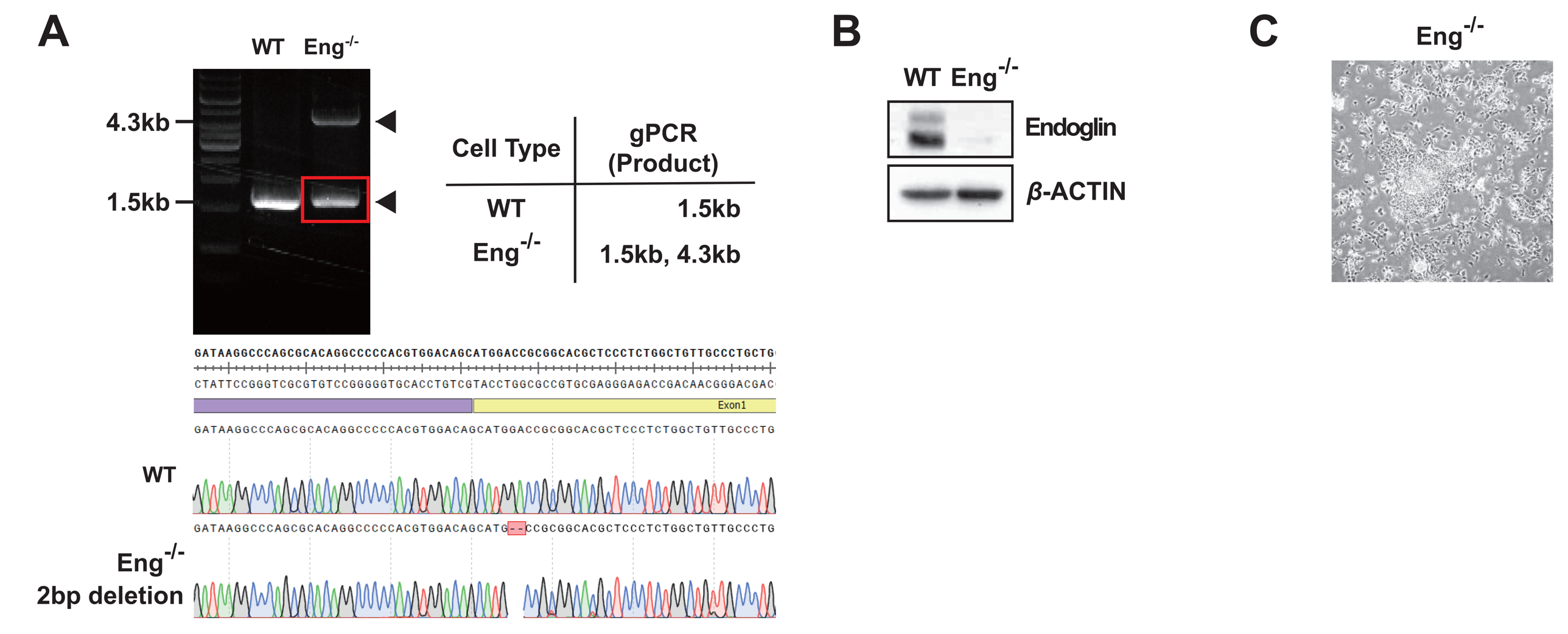


**Suppl. Fig. 1.** **Characterization of ENG^-/-^ hPSCs.** (A) Genotyping and Sanger sequencing of ENG^-/-^ cells. (B) Western blotting of *Endoglin*, showing knock-out in hPSCs. (C) Phenotype of ENG^-/-^ hPSCs. Data are means.


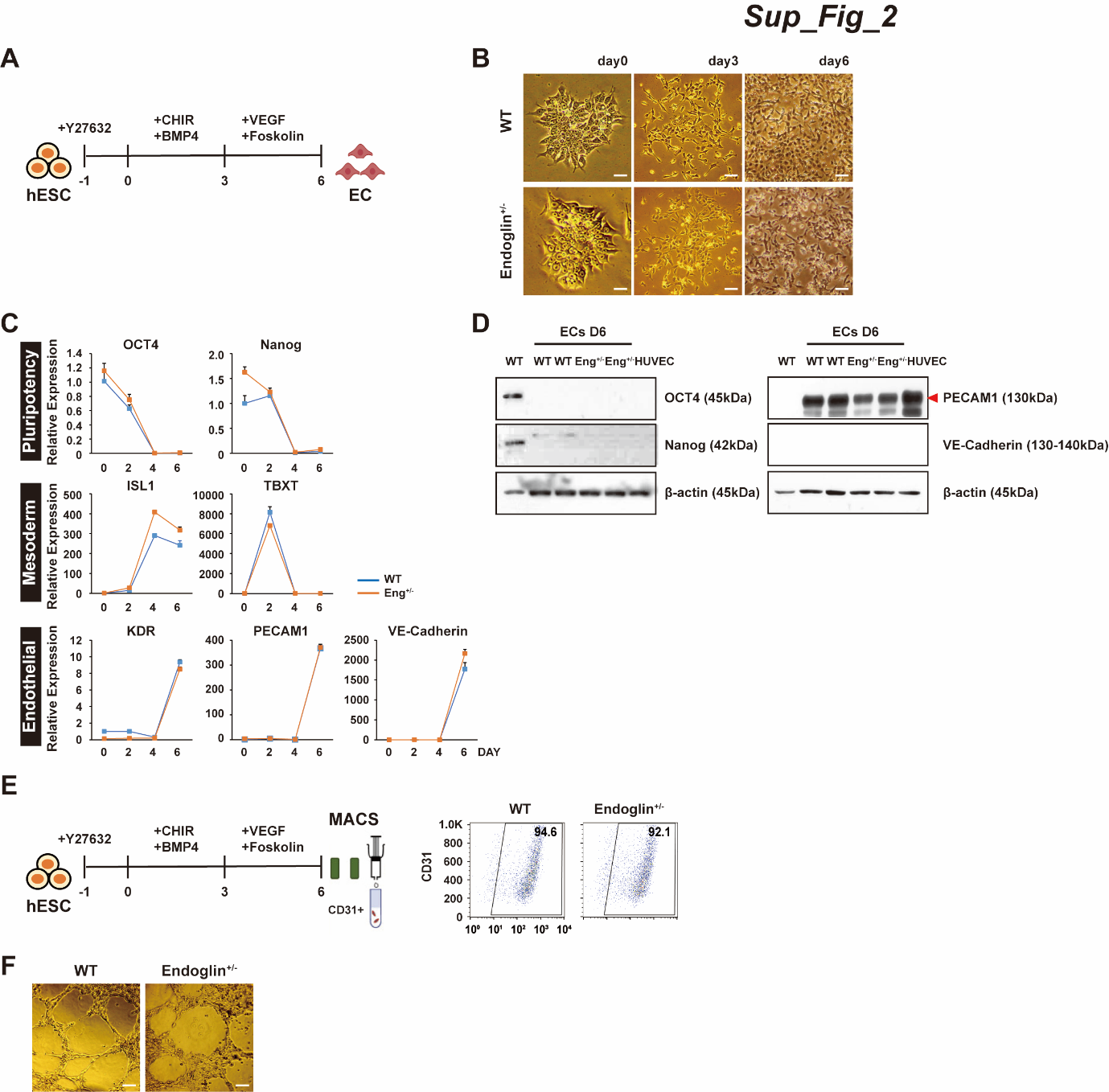


**Suppl. Fig. 2. Differentiation of ECs.** (A) EC differentiation protocol. (B) EC morphology on Days 0, 3, and 6. (C) RT-PCR analysis of pluripotency (OCT4 and Nanog), mesoderm (ISL1 and TBXT) and endothelial (KDR, PECAM1, and VE-cadherin) genes. (D) Western blotting of pluripotency and endothelial markers. (E) Scheme of MACS and FACS analyses of CD31 on Day 6 of EC differentiation. (F) Tubule formation assay of WT *vs*. ENG^+/-^ ECs.





**Suppl. Fig. 3. Differentiation of SMCs.** (A) SMC differentiation protocol. (B) RT-PCR analysis of pluripotency (OCT4 and Nanog), ectoderm (SOX2 and NESTIN), and SMC (CNN1, SM22α, and α-SMA) genes. (C) Western blotting of pluripotency and SMC markers.


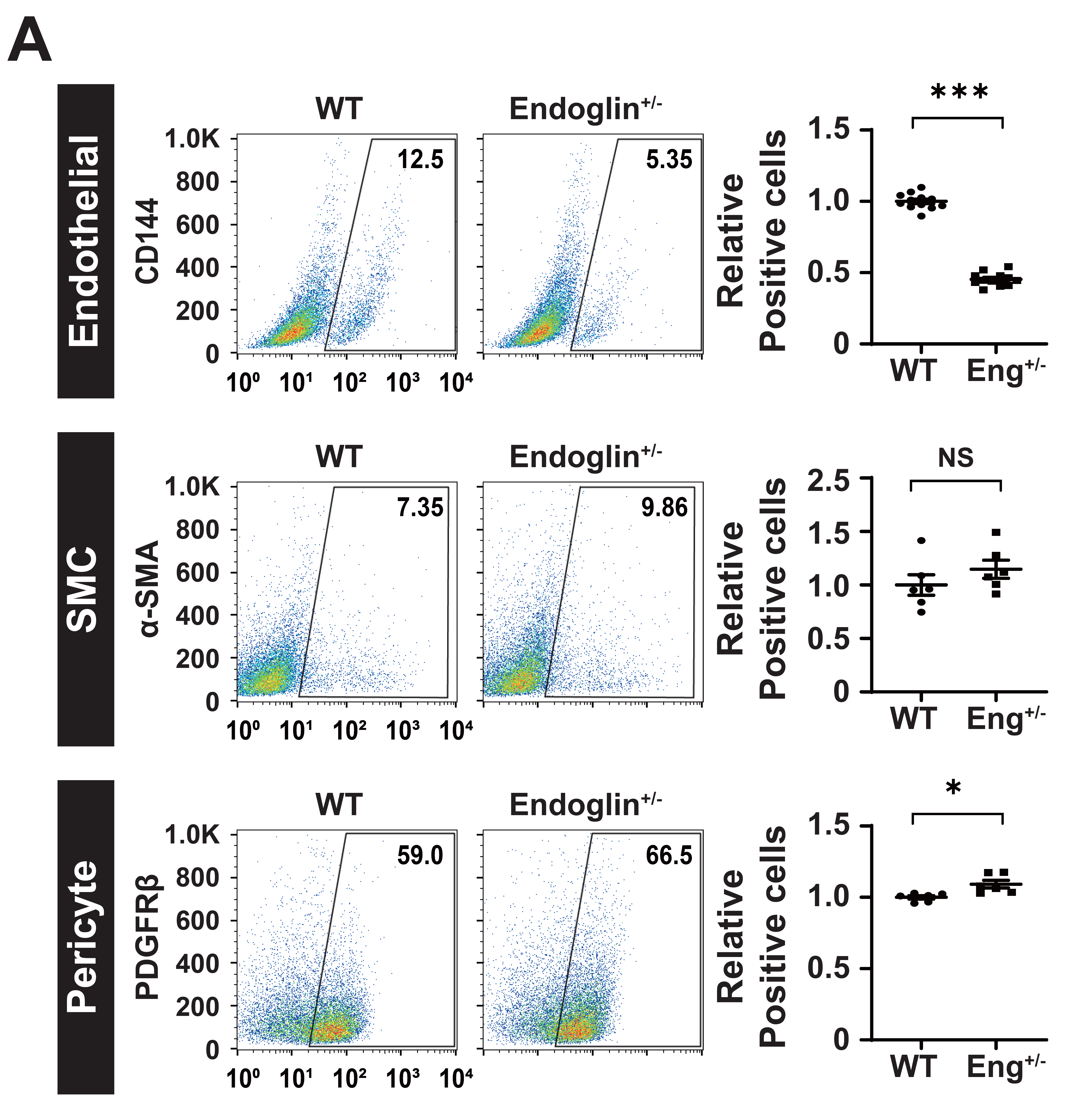


**Fig. S4.** **VEGF signaling in ECs.** (A) Western blotting analysis of the levels of downstream effectors of VEGF signaling (AKT, Erk, and p38).


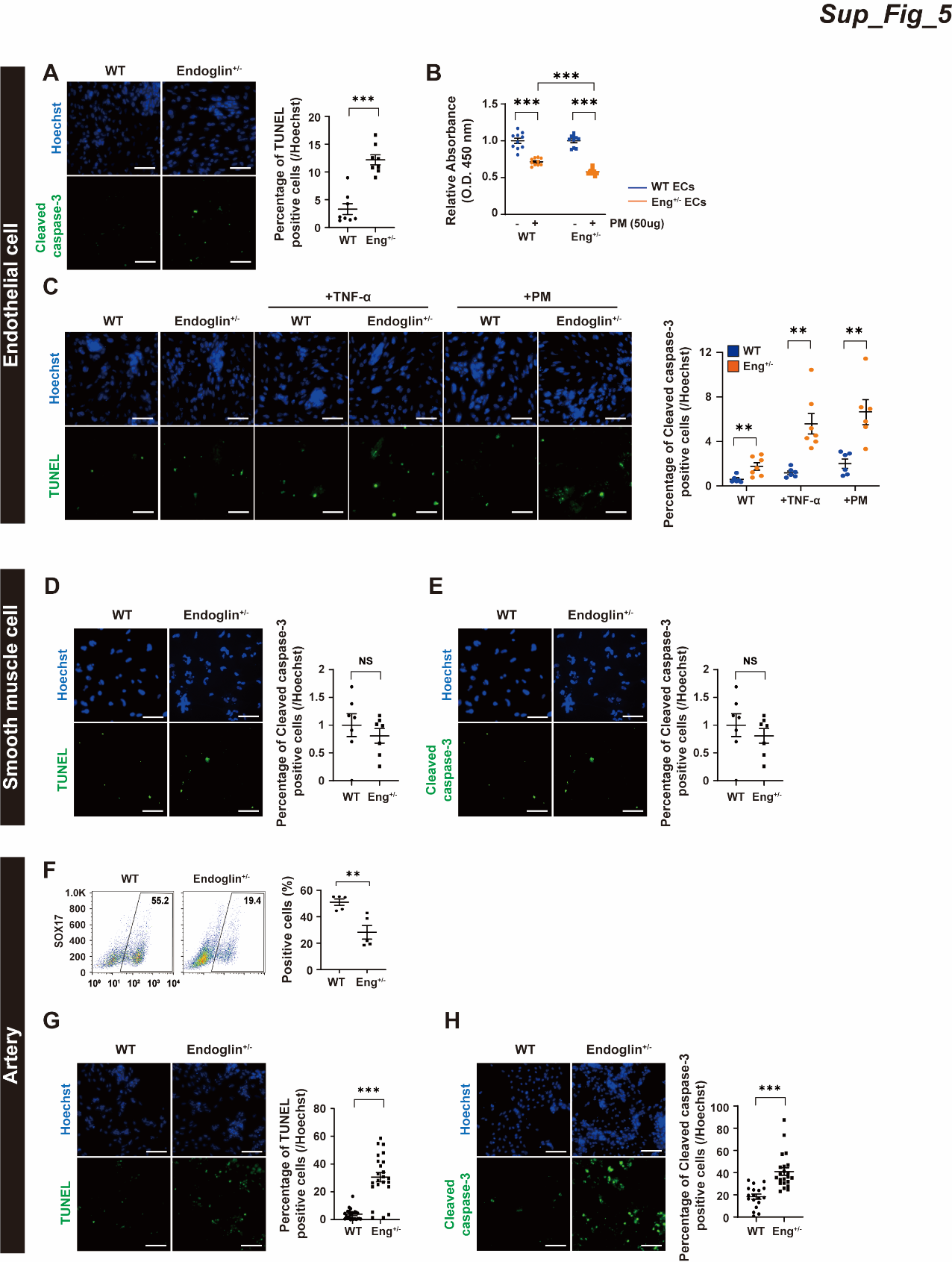


**Suppl. Fig. 5. Characterization of ECs, SMCs, and artery against WT *vs*. ENG^+/-^ clone 2.** (A) Immunocytochemical analysis of cleaved caspase-3 in WT and ENG^+/-^ clone 2 ECs (n = 8). (B) Cytotoxicity assay of WT and ENT^+/-^ clone 2 ECs treated without or with PM_2.5_ (n = 10) (C) TUNEL assay of WT and ENG^+/-^ clone 2 ECs treated without or with TNF-α and PM_2.5_. (n = 6-7) (D) TUNEL assay and (E) immunocytochemical analysis of cleaved caspase-3 in WT and ENG^+/-^ clone 2 SMCs. (F) FACS analysis of the artery marker, SOX17, in WT and ENG^+/-^ clone 2 arteries. (G) TUNEL assay and (H) Immunocytochemical analysis of cleaved caspase-3 in WT and ENG^+/-^ clone 2 arteries. Scale bars, 200 µm. (***p*< 0.01, and ****p*< 0.001). Data are means ± SEM. Values of *p* < 0.05 by two-tailed *t*-test are depicted.


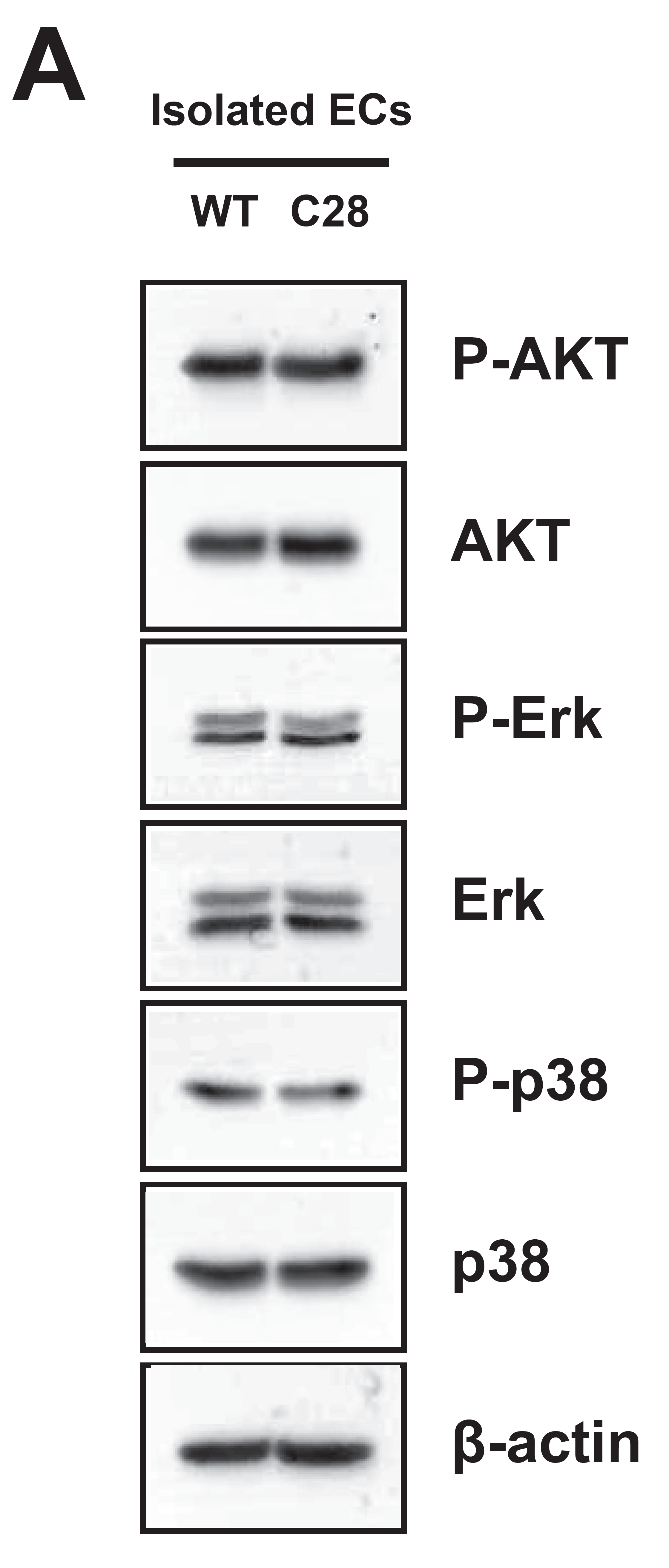


**Suppl. Fig. 6. VEGF signaling in ECs.** (A) Western blotting analysis of the levels of downstream effectors of VEGF signaling (AKT, Erk, and p38).
