# Supplementary material for: Employment of diverse in vitro systems for analyzing multiple aspects of disease, hereditary hemorrhagic telangiectasia (HHT)
Source: Cell Biosci. 2024 May 22;14:65. doi: 10.1186/s13578-024-01247-z (PMC11110195; doi:10.1186/s13578-024-01247-z)
Supplement: Supplementary file 2 — Supplementary material 2. [file 13578_2024_1247_MOESM2_ESM.docx]

**Table 1.** Primers used in qRT-PCR

| **Gene** | **Forward**  **(5'-3')** | **Reverse**  **(5'-3')** | **Accession #** | **Anealing**  **Tm (℃)** | **PCR size**  **(bp)** |
| --- | --- | --- | --- | --- | --- |
| ***OCT-4*** | GGAGAATTTGTTCCTGCAGTGC | GGAGACCCAGCAGCCTC | DQ486513 | 62 | 177 |
| ***NANOG*** | AACAGGTGAAGACCTGGTTCC | CTGAGGCCTTCTGCGTCACA | AB093576 | 63 | 102 |
| ***ISL-1*** | AGATTATATCAGGTTGTACGGGATCA | ACACAGCGGAAACACTCGAT | U07559 | 62 | 117 |
| ***TBXT*** | ATGAGCCTCGAATCCACATAGT | TCCTCGTTCTGATAAGCAGTCA | NM_003181 | 61 | 109 |
| ***KDR*** | TGATGCCAGCAAATGGGAAT | GCACCACGGCCAAGAGGCTTA | NM_002253 | 62 | 66 |
| ***PECAM-1 (CD31)*** | GAGTCCTGCTGACCCTTCTG | ATTTTGCACCGTCCAGTCC | NM_000442 | 61 | 107 |
| ***VE-CADHERIN*** | ATCAAGCCCATGAAGCCTCT | GGTCCTGCGGATGGAGTATC | NM_001795 | 62 | 282 |
| ***SOX-2*** | GAGCTTTGCAGGAAGTTTGC | GCAAGAAGCCTCTCCTTGAA | OP680447 | 61 | 190 |
| ***NESTIN*** | GAGAACTCCCGGCTGCAAAC | CTTGGGGTCCTGAAAGCTGAG | NM_006617 | 63 | 66 |
| ***CALPONIN-1*** | GTCCACCCTCCTGGCTTT | AAACTTGTTGGTGCCCATCT | D17408 | 61 | 157 |
| ***SM22α*** | CGCGAAGTGCAGTCCAAAAT | CAGCTTGCTCAGAATCACGC | D17409 | 62 | 162 |
| ***α-SMA*** | ACTGCCTTGGTGTGTGACAA | TCCCAGTTGGTGATGATGCC | J05192 | 63 | 224 |
| ***DLL4*** | ACTGCGAGAAGAAAGTGGACAGG | ACATGAGCCCATTCTCCAGGTCA | NM_019074 | 65 | 198 |
| ***NOTCH1*** | AGGACCTCATCAACTCACACGC | TCTTTGTTAGCCCCGTTCTTCAG | NM_017617 | 63 | 130 |
| ***NOTCH3*** | AGGCCATGGTCTTCCCTTAC | CAGAGCCGGTTGTCAATCTCC | NM_000435 | 61 | 119 |
| ***NOTCH4*** | ATGCGAGGAAGATACGGAGTGG | TCGGAATGTTGGAGGCAGAAC | U95299 | 63 | 112 |
| ***HES1*** | CCTGTCATCCCCGTCTACAC | CACATGGAGTCCGCCGTAA | NM_005524 | 62 | 98 |
| ***HEY1*** | GTTCGGCTCTAGGTTCCATGT | CGTCGGCGCTTCTCAATTATTC | AJ272214 | 62 | 88 |
| ***HEY2*** | GCCCGCCCTTGTCAGTATC | CCAGGGTCGGTAAGGTTTATTG | AB044755 | 61 | 80 |
| ***c-MYC*** | GGCTCCTGGCAAAAGGTCA | CTGCGTAGTTGTGCTGATGT | NM_002467 | 61 | 119 |
| ***GAPDH*** | AGCCACATCGCTCAGACAC | GCCCAATACGACCAAATCC | M33197 | 60 | 66 |
